# Physical Education

**Published:** 1860-10

**Authors:** 


### ARTICLE VII.

### *Physical Education.*

It is worthy of notice that in the period when history must record the mania for all new doctrines and isms, we have had one of the most practically valuable novelties for the restoration to health ever introduced—Dr. Winship's system of Gymnastics. We call it novelty, not in the sense of being new, but as coincident with new humbugs of every kind, aiming to get a place in the rages of the day. It is particularly noticeable for its freedom from mystical pretensions, or from the wonderful ; it is preeminently practicable and available to all. The masses of the people so long accustomed to receive blindly the arbitrary claims of the charlatan, that they hesitate to believe, indeed almost reject a matter which, with so little unintelligibility, promises to do what common understanding, when exercised, recognizes at once that it can do, that is, modify if not remedy the disease of the body—not by faith nor homeopathy ; not by mystical washings, nor by hydropathy ; not by the vegetarian temperance, but by such means as one can see are practicable, feel are useful and know to be solidly good, by at once enjoying their effects. He recognizes that hunger should be satisfied, temperance necessary, a wise indulgence in the good things of this life, a wholesome compliance to natural instincts. Drugs, manias and doctors are set down among the desperate resorts, and to replace these he supplies merely forty minutes' exercise every two days. We are not willing to go so far as the useful Doctor W. no doubt believes fully justifiable, but we feel assured that in the great majority of incipient diseases, his course will be found a practical panacea, with a better prevention of all future diseases than was ever received by a patient rising from a sickness supposed to have been cured by drugs. The practice of medicine is based upon hypotheses that our judgments are never forced to receive by practical, positive demonstration, but are simply cajoled into receiving the apparent evidence of that which must ever beg its conclusions.

The same reasoning being had would quite as conclusively prove many other inscrutable appearances as being derived from widely differing causes. Indeed, much contradictory evidence is found in the history of medicine. All who are read in medical literature will readily remember the contradictory evidence of our great leaders upon the effects and evidences of the use of strychnine. Dr. Taylor of Guy's Hospital, Mr. Letheby, and Mr. Heradeth, Prof. of Chem., affirmed that indubitable signs of the presence of strychnine were always found in the body. Dr. Christison, Mr. Nunnely and Sir Benj. Brodie could never discover any traces of it in the body. This diversity of opinion is frequent and naturally leads us to doubt the efficacy of remedies about which doctors do agree. It at least justifies a strong reliance upon remedies about which all can judge, and in the experiments of which no harm can possibly be produced. In the cause of medicine, we obviously suffer, not from our knowledge, but from our ignorance. True knowledge and science are as often misused and misapplied as they are made the instruments of vain-glory, self-love, self-praise or money-getting, tending to destroy confidence and the utility of science itself.

The system of Dr. Winship is essentially applicable to men in general, and would greatly benefit all who can be induced to adopt it, for human physiology demands a systematic exercise of the functions and powers of the body for the proper sustainment of health. Absolute inertia is nowhere found in nature. Animal life depends upon use and exercise—suspend them for any definite period and you produce disease accordingly. Every one is familiar with the results of well conducted training in all living nature, man being the particular evidence of this law dealing with higher organism. Among artizans may be found most marked proofs of this position in the higher development of the parts of the body used, when not overdone, as also of such as are not used falling to decay and waste.

We do not believe that hereditary proclivities can be entirely corrected, but that they may be kept in an inoperative state, by a judicious course of physical training, we have not a shade of doubt.

We simply protest against all generalities in medicine, knowing that each patient has differing conditions from all other patients in similar diseases. That which constitutes relief to one individual may not to another, and so we disagree with this idea that all disease, remotely or even approximately, is caused by an increased flow of blood to a part.

When such is the case, we cordially recognize the doctrine of Dr. Winship in distributing it equally all over the body, by suitable exercise, but in all atrophic diseases, tuberculosis, anemic conditions, these are not caused by a morbidly increased flow of blood to a part, but can be certainly benefited by causing an equal distribution of blood throughout the system.

Some other objectionable features may be found in this new system. All new introductions claim too much and do too little. This is not an exception. At the same time, this is better calculated to aid man in his struggle for life than any modern popular innovation.

### ARTICLE VIII.

*Artificial Dentistry, Patents, &c.* By S. M. SHEPHERD, M. D., D. D. S.

